# NEWS

**DOI:** 10.7189/jogh.06.010202

**Published:** 2016-06

## THE BILL AND MELINDA GATES FOUNDATION

➢ Bill Gates and George Osbourne, the UK Chancellor of the Exchequer, announced funding of US$ 4.3 billion over the next five years in efforts to eliminate malaria. The fund comprises US$ 345 million from the UK’s overseas budget for the next five years, with US$ 200 million in 2016 from the BMGF, with more donations to follow. In a joint letter to *The Times* newspaper, Mr Gates and Mr Osbourne highlight the global scale of malaria – 200 million cases each year, and the economic cost – mainly borne by Africa and running into billions of dollars – of lost productivity and public health expenditure. According to the World Health Organization, there were 438 000 malaria deaths in 2015, mostly in children aged under 5, and mostly in Africa. Progress is controlling malaria is being undermined in the spread of resistance to antimalarial drugs and to insecticide. (*The Guardian*, 25 Jan 2016)

➢ In November 2015, the BMGF announced an additional US$ 120 million investment in family planning over the next three years. This is a 25% increase on its current funding level, and aims to meet the Family Planning 2020 goal of giving a further 120 million girls and women voluntary access to birth control. The BMGF will continue to invest in new forms of birth control to expand the range available to women, eg, injectable methods than can be easily delivered by community health workers, or self–administered at home. (*Devex*, 11 Feb 2016)

➢ In an interview ahead of the publication of the BMGF’s annual letter, Bill and Melinda Gates highlighted how, in the wake of the Ebola tragedy, the zika virus has spurred brought a faster and more united response. Bill Gates noted that the BMGF has invested in modifying mosquitoes not to carry viruses, and in reducing their numbers, and that the same breed of mosquitoes carries dengue and zika pathogens. This year’s letter calls for young people’s involvement in tackling inequity, focusing on energy and time. It highlights the need for cheap carbon–free energy that would benefit people in developing countries, and the gap in the amount of time spent on unpaid work between men and women. This gap hampers people rising out of poverty; bringing labour–saving devices to developing countries would help free women to earn money for their families and improve health care and nutrition. (*Reuters*, 23 Feb 2016)

➢ PATH, the non–profit global health organisation, is opening a Center for Vaccine Innovation and Access, with initial funding of US$ 11 million from the BMGF. PATH will use the funding to accelerate the development and distribution of vaccines to halt deaths from preventable diseases. Currently, PATH has more than 20 vaccines at different stages of development and use, which target the world’s leading causes of child mortality – pneumonia, diarrhoea, malaria, plus diseases like polio and meningitis. PATH also plans to use the new Center to address the new threats from diseases like ebola and zika. “The new Center will bring together PATH’s expertise across the entire vaccine development and introduction process, from pre–clinical trials on novel vaccine concepts to regulatory approval and policy review, from design and conduct of field trials to innovative approaches for new vaccine development,” says Mr David Kaslow, the Center’s head. (*GeekWire*, 16 Mar 2016)

➢ The BMGF has made a US$ 5 million equity investment in Amyris Inc., a US–based bioscience company. The investment will fund work on further reducing the cost of a leading malaria treatment, focusing on the continued production of high–quality and secure supplies of artemisinic acid and amorphadiene for use in artemisinin combination therapies (ACTs), which are recommended by the WHO as the primary first–line treatment for malaria. Amyris made its artemisinic acid–producing strains available to Sanofi in 2008 on a royalty–free basis. Sanofi scaled–up this technology to produce artemisinin for ACT treatments, intending to produce enough semi–synthetic artemisinin for up to 150 million treatments by 2014, and ensure distribution on a “no profit, no loss” principle. (*Business Standard*, 12 Apr 2016)

## THE GAVI ALLIANCE

➢ GAVI has signed a US$ 5 million deal with the pharmaceutical company Merck to keep 300 000 Ebola vaccine doses ready for emergency use or further clinical trials. Merck will submit a licensing application by the end of 2017, which would help GAVI prepare a global stockpile. Early trials of the VSB–EBOV vaccine – which combines a fragment of the Ebola virus with another safer virus – suggest it may give 100% protection, although this is still preliminary. The deal was announced at the World Economic Forum at Davos, Switzerland. Isolated flare–ups of Ebola are still anticipated, and the vaccine could be important with dealing with these, as well as heading off any future epidemics. (*BBC*, 20 Jan 2016)

➢ Médecins Sans Frontières (MSF) expressed grave concern that the high cost of vaccines is not being given higher priority at the Ministerial Conference on Immunization in Africa. MSF say that the high cost of vaccines affects its ability to provide health care in developing countries, and call for pharmaceutical companies to reduce the cost of three–dose pneumonia vaccinations to US$ 5 per child, amongst others. MSF argue that lack of immunisation progress in some countries since 2013 is due to high prices – eg, vaccinations for pneumonia, diarrhoea and HPV have increased 68 times between 2001 and 2014. MSF are concerned that countries that are not poor enough to qualify for GAVI support have to negotiate prices on their own, risking their coverage rates. They call for GAVI to negotiate better deals with pharmaceutical companies. “From 2001–2014, the US has given GAVI US$ 1.2 billion in direct funding, and has pledged US$ 1 billion for 2015–18. This money can go much further if the vaccines, like Pfizer’s pneumonia vaccine, are cheaper.” (*Humanosphere*, 26 Feb 2016)

➢ Nepal passed the country’s immunisation bill in January, which aims to improve oversight of immunisation services, set higher standards for vaccine testing and usage, and change how Nepal finances its immunisation programme. Nepal currently relies on financial support from GAVI to fund 60–70% of its purchases. However, Nepal will no longer be eligible for GAVI support when it transitions from low– to middle–income status – expected by 2022, thus giving a few years to establish its own domestic financing arrangements. The new law sets out two methods for financing immunisation. First, the law commits the government to allocating funds to the National Immunization Fund, levied through general taxation. Second, health partners can contribute to a separate Sustainable Immunization Support Fund – although this will probably requires incentives such as tax exemptions to be effective. The law highlights Nepal’s commitment to immunisation, and the Chairperson of the Parliamentary Committee on Women, Children, Senior Citizens and Social Welfare, Hon. Ranju Kumari Jha, calls it “a milestone to protect child rights of getting quality immunisation service, increase country ownership and sustain the national immunisation programme by securing adequate funding.” (*healthaffairs.org*, 7 Mar 2016)

➢ Seth Berkley, GAVI’s CEO, believes that Ebola and zika have removed attention from measles. He argues that measles should be prioritised, partly because it is highly infectious and kills 115 000 people each year, and also because measles outbreaks act as an early warning system against other threats to global health security. Measles’ highly infectious nature means that outbreaks are a useful measure for gauging a health system’s ability to cope with potential global epidemics, as 90% immunisation coverage is needed to reach herd immunity, compared to 80–85% for other common diseases. If populations are under–immunised, it is likely that other vital health interventions are lacking, rendering people even more vulnerable to disease outbreaks. Mr Berkeley calls for more resources on routine immunisation, supplemented with catch–up campaigns as required. (*Devex*, 27 Apr 2016)

➢ GAVI is backing a new national drone delivery network to distribute blood supplies in Rwanda. There will be further tests planned of its suitability for a wider range of drugs, including vaccines, HIV treatments and treatments for malaria and tuberculosis. This phase will see the drones making up to 150 deliveries of blood to 21 transfusion facilities in western Rwanda – crucial for Africa, which has the world’s highest rate of maternal deaths from postpartum haemorrhaging. If successful, Rwanda’s drone network could save thousands of lives and be a model for other countries to duplicate. The project uses drones from the Californian robotics company Zipline, and the “global citizenship” art of the delivery and logistics giant UPS. “It is a totally different way of delivering vaccines to remote communities and we are extremely interested to learn if UAVs [unmanned aerial vehicles] can provide a safe, effective way to make vaccines available for some of the hardest–to–reach children,” says Seth Berkley, CEO of GAVI. (*Pharma Market Live*, 9 May 2016)

## THE WORLD BANK

➢ Ahead of a World Bank conference on how improved land management can reduce poverty and foster development, Mr Klaus Deininger, the conference organiser, argues that women’s right to land creates other benefits. These include improved health and education for children, increased household resources, and fewer child marriages as daughters are less likely to be married for financial reasons. Women with land rights tend to have bank accounts, and their financial resources can render them less vulnerable to domestic violence. In sub–Saharan Africa, women comprise more than 50% of the agricultural labour force, but fewer than 20% own farms. According to the UN World Food Programme, if women farmers had the same access to land as men farmers, global hunger could be substantially reduced. The conference will focus on women and property, with particular emphasis on gender equality and land rights – key to achieving the Sustainable Development Goals. (*Thomson Reuters Foundation*, 13 Mar 2016)

➢ Costa Rica, already considered to have one of the best health care systems in Latin America, has been granted a US$ 420 million loan to further strengthen the financial sustainability of its universal health insurance system, and the management, organisation and delivery of its services. This is in line with Costa Rica’s strategic health agenda, which was developed by the Costa Rican Social Security Administration to modernise primary health care networks. It will include the expansion of e–health and a 40% increase in screening in areas with high incidence of colon cancer. The programme will include a 25% advance to ensure progress. (*Public Finance International*, 21 Mar 2016)

➢ The World Bank announced a US$ 5 billion loan to Tunisia to support its democratic transition and economic development. Tunisia has been hit with falling tourist revenues – which account for 7% of GPD – after the Islamic militant attacks in 2015, unrest over unemployment and limited economic reforms despite wider political advances following its 2011 uprising. Economic growth was 0.8% in 2015, and is forecast to increase to 2.5% in 2016, but unemployment is 15.1% and is much higher amongst the country’s youngsters – who comprise more than 50% of the population. The loans will be used to stimulate investment and job creation, and to intensify development in disadvantaged areas. The World Bank agreed that Tunisia’s economic reforms to date are headed in the right direction, but more reforms are needed in the financial sector and to increase transparency. The International Monetary Fund and Tunisia are also in talks over a US$ 2.8 billion credit to support economic reform. (*Al Arabiya*, 25 Mar 2016)

➢ Following on from the annual spring meeting of the World Bank and IMF, 5 key themes have emerged. First, the World Bank’s track record of involuntary resettlement – which has faced severe criticism from human rights groups – was put under the spotlight, as the bank’s Inspection Panel made recommendations for better practice. Second, there were moves towards closer collaboration with other development banks, which could help close the US$ 60–70 billion infrastructure gap in Africa, amongst others. Third, in the wake of the “Panama papers”, World Bank President Jim Yong Kim emphasised how tax avoidance hinders ending poverty, and that world leaders wish to work with the Bank to track down illicit revenue flows. Fourthly, the President of the African Development Bank, Akinwumi Adesina, wants Africa’s leaders to focus more on nutrition. And finally, UN Secretary General Ban Ki–moon called for more work on addressing the root causes of conflict behind the global refugee crisis, and for the world to mobilise to ensure the safety and well–being of those crossing borders. (*Devex*, 20 Apr 2016)

➢ According to a World Bank study, South Asia could create millions of new jobs in the clothing industry by taking advantage of rising manufacturing costs in China, boosting both economic growth and job opportunities for women. With low labour costs and a growing young, working–class population, South Asia is strongly positioned to increase its share of this labour–intensive industry. Women’s participation in South Asia’s labour market is low, and increasing job opportunities for women is vital for raising marriage ages, reducing birth rates, improving nutrition and school enrollments, and stronger economic growth. However, the industry has a track record of poor working conditions – highlighted by the collapse of the Rana Plaza building in Bangladesh in 2013 – and growth opportunities will not be fully realised without closer attention to safety and improved conditions, due in part to increased scrutiny from global brands and retailers. (*Voice of America*, 29 Apr 2016)

## UNITED NATIONS (UN)

➢ The World Food Programme (WFP), a UN agency, is leading a project to boost incomes and improve food security in developing countries. It will help 1.5 million small–scale farmers across Africa, Asia and Latin America with contracts to buy their crops, signed before they are planted, to a value of US$ 750 million. It aims to enable marginalised farmers to access reliable markets – 50% of the world’s 795 million people are farmers, and in some African countries up to 90% of the population are smallholder farmers – so that farmers could move from subsistence to market–oriented production. However, critics warn that the project could fail if it does not prioritise helping poor farmers to adapt to climate change, by promoting crops which are more resilient to drought. In addition, there are concerns that farmers will be encouraged to buy hybrid seeds which require chemical fertilisers which deplete soil, and rely on regular rainfall. The WFP responded by noting that participating farmers can buy seeds from any supplier, but will receive recommendations on which seeds are best for their soil and water conditions. (*Thomson Reuters Foundation*, 10 Feb 2016)

➢ 175 world leaders gathered in New York to ratify the Paris climate deal on the world Earth Day, marking the first steps towards binding the countries to the promises they made to cut greenhouse gases. It will come into effect when the 55 countries responsible for 55% of greenhouse gases have ratified the accord, and is set to begin in 2020. China and the USA have agreed to ratify in 2016, and the EU’s 28 member countries are expected to ratify within 18 months. The agreement comes as the 2016 El Niño is believed to have caused droughts, floods, severe storms and other extreme weather patterns, and 2016 is set to break global temperature records. In welcoming the agreement, the UN Secretary–General Ban Ki–moon said “the era of consumption without consequence is over. We must intensify efforts to decarbonise our economies. And we must support developing countries in making this transition.” (*Al Jazeera*, 22 Apr 2016)

➢ April’s UN General Assembly Special Session on the World Drug Problem (UNGASS) did not lead to any radical shifts in drug policy. The central goal of the UN global drug policy is the elimination in the sale and use of illegal narcotics. The hard–line interpretation of this policy – used by most countries – does not lead to harm reduction, which underpins the UN conventions on drugs. Countries such as Mexico, Guatemala and Colombia have agreed that this approach has failed, and benefits criminals. Whilst UNGASS has not shifted from this policy, there are changes in the language around drug use, which reflects a greater focus on prevention and treatment – albeit falling short of what is required to address the estimated 400 000 drugs–related deaths each year. In moves widely seen as significant, countries such as Mexico and Canada quietly announced at the session that they are moving away from UNGASS policy by introducing their own reforms (eg, on cannabis use and legalisation). Many campaigners call for full decriminalisation of drug use, although this would not ensure the elimination of violence and corruption around black markets. In summary, whilst there was no radical changes, the session heralds some important first steps in the evolution of global drugs policies. (*Huffington Post Australia*, 2 May 2016)

➢ The UN has convened the first World Humanitarian Summit (WHS), to take place on 23–24 May, in response to the worst humanitarian crisis since World War II. One UN report found that the average length of displacement is 17 years, and another UN/World Bank report found that 90% of Syrian refugees in Jordan and Lebanon live below the national poverty line, and many are unable to legally earn money, and many children cannot access education. However, commentators have noted it is unclear what outcomes or actions the summit will produce. Médecins Sans Frontières (MSF) have pulled out of the summit, expressing concerns that the summit will not improve emergency response and reinforce impartial humanitarian aid; and nor will it make states accountable or responsible. This decision has added to the debate over creating “better aid”, and Care International emphasises the importance of addressing the demand side, as well as reactive humanitarian aid. Mr Gareth Price–Jones of Care International notes the nexus between humanitarian aid and development aid, and addressing them together could more effectively address complex, long–term crises. (*IPS*, 9 May 2016)

➢ Turkish security forces have been accused by the UN and the group Human Rights Watch of committing serious human rights violations against Turkish civilians and Syrian refugees. Turkish security forces may have deliberately shot civilians, destroyed infrastructure, carried out arbitrary arrests, and caused displacements in an ongoing military campaign against ethnic Kurdish separatists in the country’s southeast. A separate report from Human Rights Watch claim that Turkish border guards have shot and beaten Syrian asylum seekers. The UN said that many Kurdish–majority towns in the southeast have been sealed off “for weeks” and are almost impossible to access, and that there are reports of ambulances and medical staff being prevented from reaching the wounded. This is in the wake of a deal between the EU and Turkey to halt the flow of migrants to Europe, in exchange for aid and visa–free travel for Turkish citizens. (*Washington Post*, 10 May 2016)

## UNAIDS AND THE GLOBAL FUND

➢ Cambodia’s dispute with the Global Fund over travel expenses is now resolved, allowing the country to access millions of dollars in aid money to fight malaria – amidst fears over the rise of drug–resistant malaria. The dispute appears to have arisen over receipts for travel payments – these are difficult to obtain in rural Cambodia and are therefore not a requirement for government officials – and the Global Fund has agreed not to ask for these. However, travel plans must be submitted in advance, spot checks can be carried out to verify travellers’ locations, and staff will have to reimburse any “irregular” funds. The agreement means that Cambodia’s National Malaria Centre (CNM) can now access a new US$ 12 million grant, plus another (almost untouched) grant of US$ 9 million. Although welcoming the resolution, CNM’s director Dr Huy Rekol noted that Village Malaria Workers were not paid during the dispute and stopped alerting the authorities on local malaria cases, and this may be linked to some deaths from malaria. (*Phnom Penh Post*, 28 Jan 2016)

➢ The Global Fund plans to send an advance supply of antiretroviral drugs to Uganda, after the country ran out of supplies at the end of 2015. In Uganda, 1.5 million people – 1.5% of the population – are HIV positive. The shortages, which began in September 2015, affected 240 000 patients on publicly–funded treatment programmes, forcing them to modify treatment or stop outright. Private–sector clinics were unaffected. The government claimed that a weak currency and insufficient foreign exchange hindered its ability to finance drug imports. However, critics blame high election spending for the financial shortfall. The Global Fund acknowledged that the advance supply is a “short–term solution” and called for the government to mobilise resources to fill the gaps and find a long–term solution. (*Yahoo*, 25 Jan 2016)

➢ UNAIDS and Xinhua News Agency have signed an agreement to enhance global co–operation towards ending HIV–AIDS by 2030. The deal builds on an existing agreement from 2011, and new measures include strengthening collaboration in areas such as social media. The two sides will work towards this goal through in–depth co–operation, consultation and information exchange. Mr Michel Sidibé, the Executive Director of UNAIDS, said “combined with the power of media and communication, we could work together to build a legacy in promoting ending AIDS.” Xinhua President Cai Mingzhao also stated that “to end AIDS requires the joint efforts from the international community.” (*Xinhua*, 18 Mar 2016)

➢ Ahead of the UN General Assembly Special Session on the World Drug Problem, UNAIDS has released a report which shows that countries which do not adopt health– and rights–based approaches for drug–users experience no falls in HIV infections in people who inject drugs. Countries have implemented health– and rights–based approaches to drugs have reduced new HIV infections in these groups. Examples of successful programmes include the free voluntary methadone programme in China, Iran’s integrated services for the treatment of sexually–transmitted infections, injecting drug users and HIV, and a peer–to–peer outreach programme in Kenya on using sterile equipment. A key part of ending the HIV epidemic by 2020 is reaching 90% of injecting drug–users with HIV prevention and harm reduction services. This would require an annual investment of US$ 1.5 billion in outreach, needle–syringe distribution and opioid–substitution therapy in low– and middle–income countries. However, these programmes are cost–effective and deliver wider benefits, such as lower crime rates and reduced pressure on health services. (*Merh*, 17 Apr 2016)

➢ Médecins Sans Frontières (MSF) has called for governments, UN and European agencies, PEPFAR and the Global Fund to develop and implement a fast–track plan to scale–up antiretroviral treatment (ART) for countries where coverage reaches less than 33% of the population, particularly in West and Central Africa. MSF warn that globally–agreed goals to halt the HIV epidemic by 2020 will not be met without this plan. In West and Central Africa–a region of 25 countries, 75% of people who require them cannot access HIV care–equivalent to 5 million people. “The converging trend of international agencies to focus on high–burden countries and HIV ‘hotspots’ in sub–Saharan Africa risks overlooking the importance of closing the treatment gap in regions with low antiretroviral coverage. The continuous neglect of the region is a tragic, strategic mistake: leaving the virus unchecked to do its deadly work in West and Central Africa jeopardises the goal of curbing HIV/AIDS worldwide”, says Dr Eric Goermaere, MSF’s HIV referent. (*Health24.com*, 20 Apr 2016)

## UNICEF

➢ UNICEF has warned that 25 000 children are suffering from acute severe malnutrition in North Korea, and are in need to urgent treatment. It calls for US$ 18 million to support this, as part of a wider US$ 2.8 billion appeal to help 43 million children in humanitarian emergencies. In the wake of severe droughts that causes a 20% reduction in North Korea’s crop production, UNICEF needs US $8.5 million for nutrition, US$ 5 million for water and sanitation, and US$ 4.5 million for health care to help these children. There are often shortfalls in humanitarian funding for North Korea – 70% of North Koreans suffer from food insecurity, funding fell from US$ 300 million in 2004 to under US$ 50 million in 2014 – due to its restrictions on humanitarian workers and concerns over its nuclear capabilities. According to Ghulam Isaczai, the UN’s resident co–ordinator for North Korea, “[North] Korea is both a silent and underfunded humanitarian situation. Protracted and serious needs for millions of people are persistent and require sustained funding.” (*International Business Times*, 26 Jan 2016)

➢ On the eve of the International Day for Zero Tolerance of Female Genital Mutilation (FGM), UNICEF warned that growing populations in high–prevalence countries are undermining efforts to tackle the practice, which is widely regarded as a serious abuse of human rights. 50% of girls and women subjected to FGM live in Egypt, Ethiopia and Indonesia, and if current trends continue, the number of cases will increase over the next 15 years. Previously Indonesia has been excluded from UNICEF’s FGM statistics due to a lack of reliable data – its recent inclusion has led to a sharp upwards revision in the number of global FGM victims – and other countries were FGM is reported are also omitted, such as India, Oman and the United Arab Emirates. However, countries such as Liberia, Burkina Faso and Kenya have experienced steep falls in FGM cases and condemnation is growing, and UNICEF calls for accelerated efforts to eliminate the practice. (*Thomson Reuters Foundation*, 5 Feb 2016)

➢ UNICEF estimates that one–third of combatants in Yemeni’s civil war are children, on both the rebel side and troops fighting for President Abdullah Mansour Hadi. UNICEF believes that children as young as 14 are front–line fighters, despite pledges from both sides to end the practice. The massive destruction of schools and infrastructure encourages children to fight, and the rise of terrorist groups such as Isis and al–Shabaab makes negotiations over child combatants impossible. The situation in South Sudan is graver still, with 16 000 children being recruited into both sides in the country’s civil war. Anthony Nolan, one of UNICEF’s child protection specialists, says many children are driven to join by a lack of resources or a desire to seek revenge for their families, and that their recruitment threatens to prolong the conflict for future generations. (*The Independent*, 8 Feb 2016)

➢ On the 5^th^ anniversary of the Syrian civil war, a UNICEF report shows the resultant refugee crisis, with over 2.4 million Syrian children living as refugees outside their country, 200 000 live as refugees within Syria – and a further 306 000 children were born as refugees. Over 250 000 people have died in the conflict – at least 400 children were killed in 2014. And now, twice as many people live in areas under siege or otherwise hard–to–reach compared to 2013, and 2 million of those cut off from help are children, with UNICEF reporting children suffering from extreme malnutrition or death from starvation. There are concerns over the increases in recruitment of child soldiers – both boys and girls, and children have reported being beaten, indoctrinated and forced to commit violence. The psychological effects of living under siege are also devastating. “Children living under siege almost have to re–learn what it’s like to be a human being,” says Mr David Nott, a trauma surgeon who has worked in Syria. (*Business Insider*, 14 Mar 2016)

➢ According to UNICEF, more than 700 million women were married before their 18^th^ birthday, and Bangladesh has the world’s second highest rate of marriage of girls aged under 15, after Niger. However, a study by the New York–based Population Council shows that child marriage fell by 31% when girls are educated or took classes in critical thinking and decision–making, with further falls when girls received job skills training. In Bangladesh, 75% of girls marry before they are 18 years old. “In Bangladesh, limited evidence exists on what works to delay child marriage. These results are a major leap forward,” said Ann Blanc, Vice President of the Population Council. (*Thomson Reuters Foundation*, 23 Mar 2016)

## WORLD HEALTH ORGANIZATION (WHO)

➢ A WHO report published ahead of the first Ministerial Conference on Immunisation in Africa shows that Rwanda’s immunisation coverage is 99%. This success is attributed to improving routine immunisation and the introduction of new vaccines. Dr Matshidiso Moeti, the WHO regional director for Africa, noted that Africa has increased vaccination coverage from 64% in 2004 to 79% in 2014. However, she urged further action from governments at the conference, because only 9 countries have immunisation coverage of 80% or higher, and 1–in–5 children in Africa do not receive basic vaccinations. “We have the tools, we need to save children’s lives, and all we need is the political will and financial support to deliver,” she said. Currently, GAVI funds immunisation in 70% of African countries, but as more African countries move from low– to middle–income status they will be ineligible for GAVI support, so must prepare to meet immunisation costs from their own budgets. (*allafrica.com*, 26 Feb 2016)

➢ The WHO, in its role as pharmaceutical watchdog in markets with inadequate regulation, has suspended its approval of tuberculosis drugs made by India’s Svizera Laboratories. The company is a major supplier to developing countries, and the move follows concerns over its manufacturing and quality standards. The WHO also recommended that batches of medicine already on the market should be retested by independent experts, and that supplies may need to be recalled. The company disagreed with the WHO’s decision (which follows earlier warnings on standards in Svizera Laboratories, including dirty surfaces, black mould in a cleaning area, poor hygiene and inadequate record keeping), claiming the WHO had ignored evidence that Svizera’s operations were up to standard. India’s pharmaceuticals industry supplies cheap copies of generic drugs, but in recent years it has been beset by problems over the quality of its products. (*medicaldaily.com*, 19 Mar 2016)

➢ The WHO’s zika response differs markedly from its 2014 response to the Ebola outbreak. The WHO quickly flagged zika as a public health emergency – despite significantly fewer deaths – it took 5 months and nearly 1000 deaths before it declared Ebola a “public health emergency of international concern.” Although the faster response – intended to jump–start scientific research, vaccine and treatment development, and mosquito control, may partly be caused by a wish to act quickly after criticisms over Ebola – the overall picture is more nuanced. For example, the WHO’s regional office for the Americas (PAHO) had more expertise on zika compared to the Ebola expertise within the WHO’s regional office in Africa, and PAHO came under pressure from the USA – which is more likely to be affected by zika than Ebola – to act decisively. Finally, the impact of zika can be presented in distressing images of newborn babies with microcephaly, whereas Ebola affected wider swathes of society, thus making it harder to press for action for a single group. (*Chicago Tribune*, 5 Apr 2016)

➢ A WHO–led analysis published in *The Lancet Psychiatry* shows how the global failure to tackle depression and anxiety costs US$ 1 trillion each year in lost productivity and causes “an enormous amount of human misery.” It found that without scaled–up treatment, 12 billion working days – 50 million years of work – will be lost to depression and anxiety disorders each year up to 2030. Scaling–up treatment would cost US$ 147 billion, meaning that every US$ 1 invested in treatment would lead to a US$ 4 return in better health and ability to work, and the authors argue that both developing and developed countries should improve mental health care. The study notes that common mental health conditions are increasing – the number of people suffering with depression and/or anxiety rose from 416 million in 1990 to 615 million in 2013. 10% of the world’s population is affected, and mental disorders account for 30% of the global burden of non–fatal diseases. Treating these disorders would help the world meet the SDG of reducing premature deaths from non–communicable diseases by 33% by 2030. War and humanitarian crises increase this urgency, as the WHO estimates that up to 20% of people suffer from depression and anxiety during emergencies. (*The Guardian*, 12 Apr 2016)

➢ In April, the WHO launched its global strategy to combat leprosy, with the overall aim of reducing to zero the number of children diagnosed with leprosy. Although the disease prevalence rates for leprosy fell to below 1 per 10 000 population in 2000, worldwide there are still 213 899 new cases a year – and India, Brazil and Indonesia account for 81% of these cases. Key interventions to combat leprosy include targeting detection amongst higher–risk groups via campaigns in highly endemic areas, and improving health care coverage for marginalised groups. Early detection, especially amongst children, is essential for reducing disabilities and transmission. (*livemint.com*, 21 Apr 2016)

